# Three year experience with the cochlear BAHA attract implant: a systematic review of the literature

**DOI:** 10.1186/s12901-016-0033-5

**Published:** 2016-10-01

**Authors:** Panagiotis A. Dimitriadis, Matthew R. Farr, Ahmed Allam, Jaydip Ray

**Affiliations:** 1 Department of Otolaryngology, Sheffield Teaching Hospitals, Sheffield, UK; 2 Department of Otolaryngology, Mansoura University Hospitals, Mansoura, Egypt

**Keywords:** BAHA Attract, Transcutaneous bone conduction device, Hearing loss

## Abstract

**Background:**

Bone conduction devices are widely used and indicated in cases of conductive, mixed or single sided deafness where conventional hearing aids are not indicated or tolerated. Percutaneous bone-conduction devices gave satisfactory hearing outcomes but were frequently complicated by soft tissue reactions. Transcutaneous bone conduction devices were developed in order to address some of the issues related to the skin-penetrating abutment. The aim of this article is to present a systematic review of the indications, surgical technique and audiological, clinical and functional outcomes of the BAHA Attract device reported so far.

**Methods:**

A systematic computer-based literature search was performed on the PubMed database as well as Scopus, Cochrane and Google Scholar. Out of 497 articles, 10 studies and 89 reported cases were finally included in our review.

**Results:**

The vast majority of implanted patients were satisfied with the aesthetics of the device scoring highly at the Abbreviated Profile of Hearing Aid Benefit, Glasgow Benefit Inventory and Client Oriented Scale of Improvement. Overall, hearing outcomes, tested by various means including speech in noise, free field hearing testing and word discrimination scores showed a significant improvement. Complications included seroma or haematoma formation, numbness around the area of the flap, swelling and detachment of the sound processor from the external magnet.

**Conclusions:**

The functional and audiological results presented so far in the literature have been satisfactory and the complication rate is low compared to the skin penetrating Bone Conduction Devices. Further robust trials will be needed to study the long-term outcomes and any adverse effects.

## Background

The notion of bone conduction hearing was mentioned as early as the second century AD by Claudius Galenus [1]. Its principle is that sound can be transferred to the inner ear by skull vibrations, bypassing the external and middle ear. Bone conduction devices (BCD) are commonly used in cases of single-sided deafness or conductive/mixed hearing loss where conventional hearing aids are not indicated or tolerated. Conventional BCD [2] were developed in the early 20th century and included a sound processor attached to spectacles or headbands [3]. Disadvantages of these devices included problems with the skin and soft tissue under the transducer as well as tension headaches due to a high static pressure of about 2 N [4], sound attenuation due to soft tissue interposition especially in frequencies above 1 kHz and issues with feedback [4]. Implanted BCD transmit sound vibrations directly to skull and were developed to overcome some of the issues mentioned above. They are divided into percutaneous (skin penetrating) and transcutaneous (non-skin penetrating) types.

The Bone Anchored Hearing Aid (BAHA®) was the first available percutaneous BCD. It is a semi-implantable under the skin BCD coupled to the skull via an abutment to a titanium fixture. Presently, there are two companies that manufacture the percutaneous BCD: the Swedish Cochlear Bone Anchored Solutions AB, Mölnlycke, that manufacture the BAHA® and the Danish Oticon, which manufacture the Ponto. Their sound processors continually improve offering higher output capability, improved transduced technology and better fitting procedure. To date, more than 150,000 hard of hearing individuals use BAHA [4–6].

Problems associated with these devices include: wound dehiscence, recurrent soft tissue reactions and infections around the abutment are commonly reported (range 8–59 %) which can be daunting both for the patient and the surgeon and can occasionally lead to revision surgery (range 5-42 %) [2, 7]. Implant loss rate is reported to be 8.3 %; and it is even higher in the paediatric population and individuals with learning disabilities [8]. Aesthetic appearance is also a relative drawback and it is therefore often not widely acceptable people in adolescence or by people from different cultural backgrounds [9].

The Bonebridge from MED-EL, Innsbruck, Austria is a direct-drive BCD that is non-skin penetrating. Its transducer is completely implanted and the external processor is attached to the skin by retention magnets in the implanted unit [10].

The skin-drive or transcutaneous BCD transmit sound vibrations through the skin and were developed in order to address some of the issues related to the presence of the skin-penetrating abutment. Hugh and colleagues developed and implanted the first transcutaneous BCD (Xomed Audiant) and the complication rate dropped significantly [11]. It was soon taken out of the market due to poor clinical and audiological outcomes [12].

Following on from this concept, the Sophono device was developed by Siegert under the name Otomag and has been available since 2006 [13]. It has two magnets implanted to the skull by five titanium screws. It uses a larger contact area, designed to reduce skin pressure, which in turn might lead to flap problems. When the skin flap thickness is more than 6 mm, thinning is recommended [14]. A retrospective study on 20 patients with aural atresia implanted with Sophono, found an average improvement of 28.6 dB HL on Pure Tone Audiometry (PTA) and 61.6 % in speech recognition threshold (SRT) scores compared to the unaided condition [15]. Similar audiometric results were presented in studies by Magliulo et al. [16] and O’Niel et al. [17]. In O’Niel’s study, skin problems following fitting were noticed in 36 % of the patients and included swelling, irritation, infection, or pain following prolonged use of the device [17].

The BAHA Attract was launched in 2013 and so far more than 200 patients have been implanted [6].

This device uses a single magnet that is attached to the skull with a single titanium fixture. The sound processor is attached to a corresponding external magnet with a soft pad that is used to distribute pressure over the contact area and decrease skin sensitivity. The innovation in this BCD is that in cases of conversion to Attract, a previously fitted osseointegrated fixture can be used to replace the abutment with an implant magnet. We present here a systematic review of the literature.

## Methods

A systematic computer-based literature search was performed on the PubMed database as well as Scopus, Cochrane and Google Scholar. We also searched the grey literature and the manufacturer’s leaflets and publications. For each search we used the following free-text search terms: Term A was ‘BAHA’ or ‘transcutaneous’ and Term B was ‘Attract’ or ‘hearing’.

### Inclusion and exclusion criteria

We have included publications that met the following criteria:

1. Reports on patients that underwent BAHA Attract implantation
2. Published in the English language

We have excluded publications that were:

1. Book chapters, letters to the editor and editorials
2. Publications that were relevant to other transcutaneous devices but BAHA Attract
3. Publications from earlier than 2013 (i.e. before the BAHA Attract was commercially available)

## Results

### Search results

**Fig. 1:**
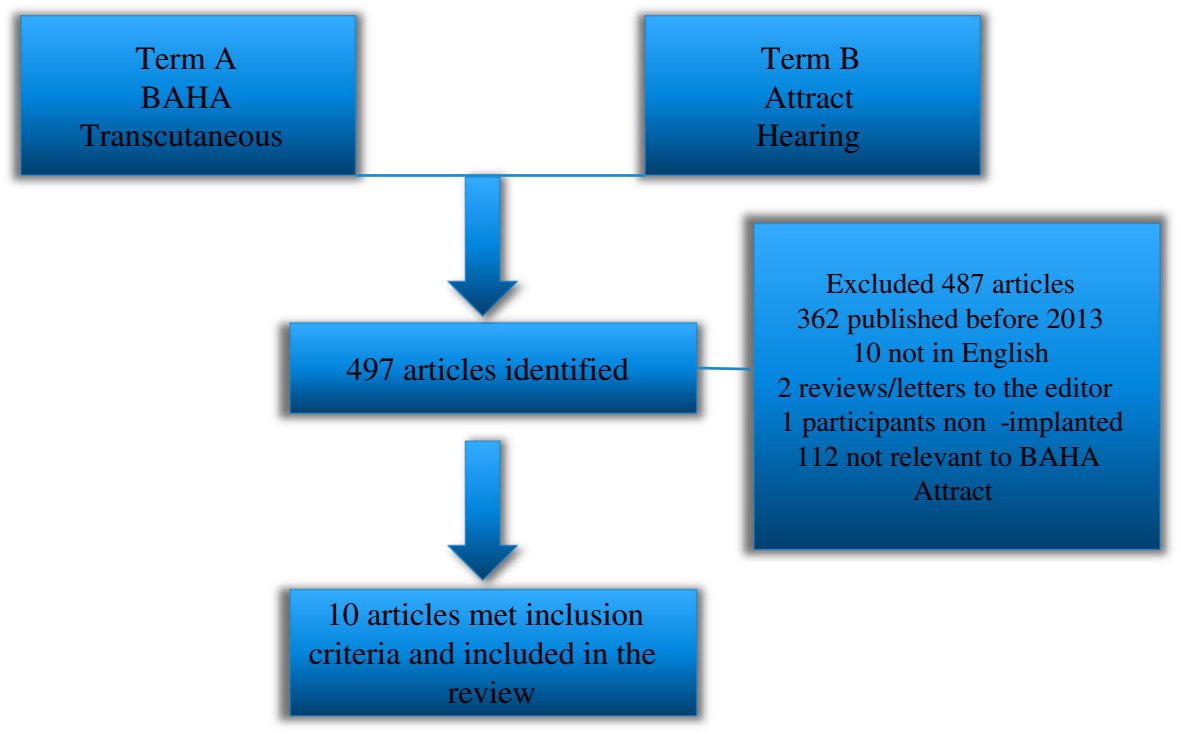
Illustration of the paper selection process

Our search strategy on PubMed revealed 497 articles. After the eligibility assessment 487 publications were excluded. In total, 10 studies were included in the review. Figure 1 illustrates the paper selection process.

### Audiological and otological indications

According to the manufacturer, patients with unilateral or bilateral conductive hearing loss (CHL), especially those with an air-bone gap of more than 30 dB would benefit from an Attract system with good hearing outcomes. In cases of mixed hearing loss, patients with a greater air-bone gap (>30 dB) would benefit more from an Attract system than an air conduction hearing aid. Regarding the sensorineural element of hearing loss, a BAHA Attract could compensate for up to 45 dB HL. Finally patients with singe-sided deafness [and low transcranial attenuation] would be able to hear due to crossing over of vibrations to the healthy cochlea and able to localise sounds better. In cases of large transcranial attenuation or moderate mixed hearing loss the patients would most likely benefit more from other hearing aid solutions [18]. Patients with the following Ear Nose and Throat (ENT) conditions would benefit from an Attract system: congenital malformations, ear canal stenosis, discharging ears with or without mastoid cavity, previous ear surgery and syndromic hearing loss (such as in Goldenhar or Treacher Collins) [18]. Of course each case should be assessed in its own merits.

**Fig. 2:**
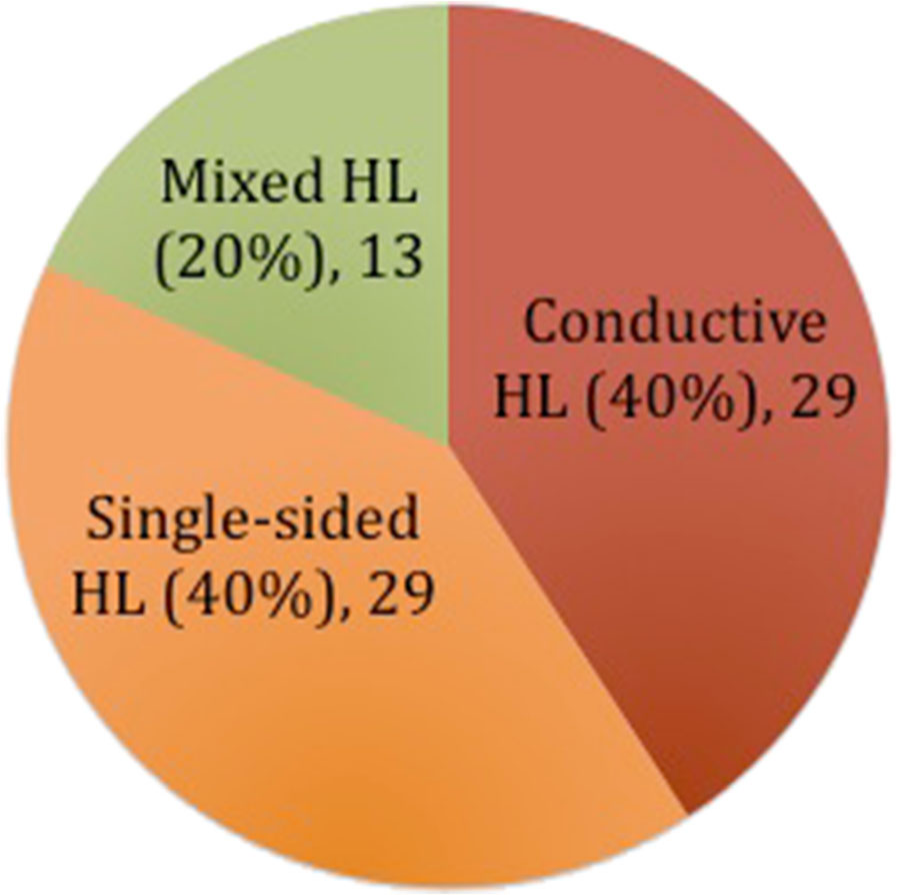
Summary of the audiological indications for implantation of BAHA Attract in the literature

**Fig. 3:**
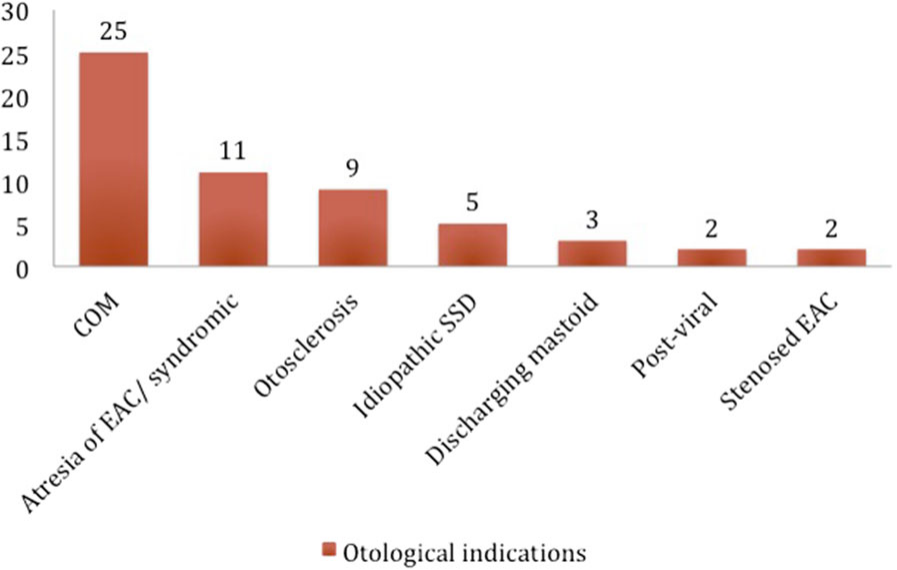
Summary of the otological indications for implantation of BAHA Attract in the literature

Figures 2 and 3 summarize the audiological and otological indications for implantation of BAHA Attract respectively, based on the cases that were found in the published studies. Table 1 includes the demographics of the patients included in the study as well as otological and audiological indications per study.

**Table 1:** Patients’ demographics, audiological and otological indications for surgery

Study	No of patients	Gender	Mean age (years), age range	Audiological indications	Otological indications
Baker 2015 [23]	6	4 M, 2 F	10.7 (5–15)	5 Unilateral SNHL 1 CHL	N/A
Gawecki 2016 [19]	20	7 M, 13 F	49.8 (25–67)	11 Bilateral Mixed HL 1 Bilateral CHL 8 Unilateral SNHL	8 COM 3 Atresia of EAC 8 otosclerosis 1 post-mumps
Deveze 2015 [6]	1	1 M	65	Unilateral SNHL	Post Ramsey-Hunt
Iseri 2014 [24], Iseri 2015 [9]	16	6 M, 10 F	28 (5–52)	N/A	14 COM 2 Atresia of EAC
Marsella 2015 [25]	3	N/A	25 (8–44)	3 CHL	2 Atresia of EAC 1 post-mastoidectomy
Clamp 2015 [2] Briggs 2015 [22]	27	12 M, 15 F	47.5 (range N/A)	17 CHL 10 Unilateral SNHL	N/A
Powell 2015 [26]	6	N/A	16 (8–46)	2 Bilateral CHL 3 Unilateral CHL, Contralateral Mixed HL 1 Unilateral SNHL	1 SSD 2 LVAS and Mondini 1 Atresia of EAC 1 Meatal stenosis 1 primary ciliary dyskinesia
Carr 2015 [26]	10	5 M, 5 F	45.8 (21–60)	7 CHL 3 Unilateral SNHL	5 COM 1 Otosclerosis 1 Meatal stenosis 3 SSD

CHL: Conductive Hearing Loss, COM: Chronic Otitis Media, EAC: External Auditory Canal, HL: Hearing Loss, N/A: Not available, No: number, M: Male, F: Female, SNHL: Sensorineural Hearing Loss, SSD: Sudden Sensorineural Deafness

In particular, out of the 89 patients included in this study, 17 (19.1 %) were children under the age of 16. From the available audiological data in the paediatric population, 5 (55.6 %) had unilateral SNHL and 4 (44.4 %) had CHL (2 had bilateral CHL and 1 contralateral mixed HL). In the adult population, 27 (45 %) had CHL, 22 (36.7 %) had unilateral SNHL and 11 (18.3 %) had mixed hearing loss (bilateral). Otological problems seen in the children included atresia of the external auditory canal (EAC) (36.4 %), COM (27.3 %), EAC stenosis (9.1 %), large vestibular aqueduct and Mondini dysplasia (18.2 %), ossicular abnormality (9.1 %) primary ciliary dyskinesia (9.1 %). In the adults, the majority had COM (53,3 %), followed by otosclerosis (20 %), single sided deafness (8.9 %), atresia (8.9 %), post-viral infection (4.4 %), EAC stenosis (2.2 %) and post-mastoidectomy (2.2 %).

### Evaluation of candidates

The air bone gap in the candidate’s conductive or mixed hearing loss is a good indicator on whether they would benefit from an Attract system. So, a proper audiological evaluation including PTA, speech audiometry and sound field testing, is essential in the patients’ workup. It is also important for the patients to try the Attract in different acoustic environments; this can be done by supplying them with a BAHA on a softband that that they can use for a few weeks. An unnecessary, costly procedure can be prevented that way, if the candidates do not perceive any benefit from the trial.

### Surgery

Surgery can be performed under local or general anaesthesia. Gawecki et al. (2016) performed 17 out of 20 cases under local anaesthesia and suggested that it is feasible in most adults [19]. Different implant centres’ incision site might differ slightly from that described in the company’s surgery guide [20] but most follow the typical C-shaped incision described in the surgery guide. The incision sites as well as mean surgery time and range per centre are included in Table 2. The site of the implant is marked pre-operatively; the superior edge of the processor is 5-7 cm posterior to the ear canal at the level of the temporal line. It is essential that the sound processor does not touch the pinna. A dot of methylene blue dye is injected deep at the centre of the implant site to aid correct placement of the fixture once the flap is raised. Before infiltration of local anaesthesia, skin thickness is measured in several positions of the planned implant site. If the soft tissue is thicker than 6 mm, soft tissue reduction is required to ensure adequate sound transmission [21]. In a study by Briggs et al. [22], 3 out of 5 patients with flap thickness >6 mm, had insufficient magnetic retention, despite flap thinning. A 100^0^ to 120^0^ C-shaped incision is made 15 mm away from the marked area, down to periosteum and a full-thickness scalp flap is raised. Once adequate dissection is adequate so that the magnet template can be placed in a satisfactory position, a cruciate incision is done in the periosteum, which is raised to expose enough bone for the implant flange. A bone bed indicator can be used to determine whether the surrounding bone requires polishing. Drilling follows at an angle perpendicular to the bone surface, which aims to minimise the need for bone polishing later in the procedure. Once the fixture is in situ, the magnet is screwed into the implant and tightened to 25 Ncm using the torque wrench provided. The wound is closed in layers and a head bandage is applied for 1 to 2 days. A waiting period of 4–6 weeks for osseo-integration to take place is necessary before loading of the sound processor. A BAHA softwear pad is placed in between the skin and the external magnet and provides load distribution over the entire surface of the contact area [2].

**Table 2:** Surgery time, incision, complications and their management

Study	Mean surgery time in minutes, (range)	Surgical incision	Complications - Management	Outcome
Baker 2015 [23]	N/A	As per manufacturer	1 seroma - Needle aspiration 1 device detaching	1 Resolved 1 Patient not using device
Gawecki 2016 [19]	44 (30–60)	As per manufacturer	2 Haematoma – Compression bandage	Resolved
Deveze 2015 [6]	N/A	Anterior based flap	None	
Iseri 2014 [24], Iseri 2015 [9]	46 (35–65)	Anterior based flap	1 Haematoma – Aspiration 1 Erythema – Reduced magnet strength 3 Erythema and pain – Reduced magnet strength	Resolved
Marsella 2015 [26]	N/A	As per manufacturer	1 swelling soft tissue - Antibiotics	Resolved
Clamp 2015 [2] Briggs 2015 [22]	45 (range N/A)	As per manufacturer	4 Mild erythema 4 Pain – reduced strength of magnet in 1 patient	Resolved
Powell 2015 [26]	N/A	N/A	1 Device detaching despite stronger magnet 1 Sound processor detaching from external magnet plate	N/A
Carr 2015 [27]	57 (40–80)	Inferior based flap	8 Numbness of scalp	None

*N/A* Not available

### Outline of studies and audiological outcomes

Table 3 depicts the study design as well as outcome measures and outcomes per study. Baker et al. (2015) [23], in their study performed pre-operative audiometry using inset of supra-aural headphones and compared with soundfield post-operatively. Masking was applied to the non-test ear. In average PTA thresholds were improved by 41 dBHL and speech reception thresholds by 56 dBHL. However, measuring hearing thresholds by different means (inset or supra aural headphones vs. soundfield) can affect accuracy of statistical analysis. Post-implantation audiometric data were missing from one child as the magnet was not strong enough to hold the sound processor. Gawecki et al. (2016) [19] reported on their series of 20 adult patients who underwent BAHA Attract implantation. They divided their patients in two groups, namely Group A: 11 patients with bilateral mixed or CHL and Group B: nine patients with unilateral deafness. The postoperative audiometric evaluation that was performed in 17 (85 %) patients, included speech in noise only and revealed a mean gain of 32.9 %. Iseri et al. (2015) [9] presented the results of a multi-centre study that aimed to compare BAHA Attract with percutaneous bone conduction implants. The BAHA Attract group consisted of 16 patients. Some preliminary results on 12 of them were already published in 2014 [24]. During surgery, bone polishing was required in 5 patients and soft tissue reduction in 4 patients. Post-operatively, the hearing thresholds and SRT were significantly improved (P < 0.05) when the bone conduction implant was on than without it. A between group comparison revealed a significant difference in the SRT results in favour of the percutaneous BCI group. Marsella et al. (2015) [25] reported on their experience of 3 patients implanted with BAHA Attract. The mean gain on PTA was 25 dB. A better gain was seen in the central frequencies and lower gain in the lower (250Hz) and higher frequencies (4 kHz). The SRT post-operatively was 100 % for each patient, with a mean gain of 63 %. Clamp and Briggs (2015) [2] presented some initial results from 8 patients implanted in Melbourne, Australia. This was part of a multicenter study; the other centres were in Santiago, Chile; Haifa, Israel and Hong Kong, China. A subsequent study was published later in 2015 [22] that included another 19 patients. Free field hearing testing showed a mean gain of 18.4 dB HL over the 4 central frequencies. Mean improvement in SRT in quiet was 50 % at 50 dB SPL, 46.4 % at 65 dB SPL and 24.2 % at 80 dB SPL. There was statistically significant improvement in Speech in noise Ratio of 15 dB (SD: 12.8 dB) compared to unaided hearing and 3.8 dB (SD: 7 dB) compared to soft band. For the Australian arm: Mean speech discrimination (monosyllabic words in quiet) score gain with BAHA Attract was 40.7 dB. Speech discrimination in noise was also improved (mean signal to noise difference gain of 10.6 dB). Pure Tone Audiometry results were not available and masking was applied to the contralateral ear at all conditions. Powell et al. (2015) [26] published their results on a study that compared outcomes between 6 patients with BAHA Attract and 6 that were implanted the Sophono Alpha 1. They concluded that both systems improved audiological outcomes and there was no statistically significant difference in aided thresholds or speech discrimination scores between the two devices. Mean unaided PTA was 60.8 dB HL and mean aided PTA 30.6 dB HL. Most gain was noticed at the lower and mid frequencies. At 55 dB, unaided SRT were around 18.5 %, but when aided, they improved to around 87 %. Mean speech perception score gain at 55 dB was 70 %. Carr et al. (2015) [27] reported on 10 patients who were implanted the BAHA Attract device. They performed word discrimination scores (WDS) in 3 of the patients with CHL using Boothroyd sentences. When aided, there was an increase in WDS of 50 % at 30dBA (from 0 % to 50 %), and 56 % at 50 dBA (32 % to 88 %), which was not statistically significant. Finally, Deveze et al. (2015) [6] reported on one case where a percutaneous bone conduction implant was changed to a BAHA Attract due to recurrent episodes of skin reactions around the abutment (Holgers Grade 3) that failed to improve despite having a longer abutment fitted and local treatment. The initial procedure involved soft tissue reduction. Upon removal of the abutment an interval of 2 months was kept for the skin to heal before re-operating. The authors, concerned about the skin quality and further pressure to skin by the magnet, used a superficial fascia temporalis flap that was stitched around and sheltered the magnet. Audiological results were not presented however the patient reported a decrease in the output compared to the previous percutaneous device. It is commonly accepted that the hearing gain with BAHA Attract is lower than the percutaneous BAHA, therefore they are best used in patients with normal or mildly affected cochlear function. If the hearing deteriorates (e.g. due to aging) conversion to a percutaneous BAHA device should be considered and is a straightforward procedure since there is no need to replace the fixture [2].

**Table 3:** Study design, Outcome measures and results

Study	Study Design	Outcome measures	Results (Mean improvement)
Baker 2015 [23]	Retrospective case series	Soundfield testing: PTA and SRT	PTA: 41 dB HL SRT: 56 dB HL
Gawecki 2016 [19]	Prospective cohort study	QoL questionnaires: GBI, APHAB, BAHU Free field speech in noise audiometry	APHAB: 23.5 % improvement GBI: 29.6 % improvement BAHU: “Good” or “very good” by 85 % of patients Speech in noise: 32.9 %
Deveze 2015 [6]	Case report	N/A	N/A
Iseri 2014 [24], Iseri 2015 [9]	Multicentre retrospective cohort study	Free field PTA and SRT QoL questionnaires: GBI	PTA: 27.3 dB HL SRT: 24 dB HL GBI: 40.5
Marsella 2015 [25]	Prospective case series	Free field PTA and SRT	PTA: 25 dB HL SRT: 63 %
Clamp 2015 [2] Briggs 2015 [22]	Multicentre prospective cohort study	Free field PTA and SRT Speech in noise audiometry QoL questionnaire: APHAB	PTA: 18.4 dB HL SRT: 50 dB HL at 50 dB SPL Speech in noise: 15 dB HL APHAB: significant improvement p < 0.05
Powell 2015 [26]	Cross-sectional cohort study	Free field PTA and SRT QoL questionnaires: Bone Anchored Hearing Devices questionnaire	PTA: 30.2 SRT: 72.5 Bone Anchored Hearing Devices Questionnaire: mean score 9.7/10
Carr 2015 [27]	Retrospective cohort study	Free field speech discrimination QoL questionnaires: GBI, COSI	Speech discrimination: 56 % at 50 dBA GBI:82 % and 91 % (for previously aided vs not-previously aided patients) COSI: 86 % of patients could hear in background noise 95 % of the time

*APHAB* Abbreviated Profile of Hearing Aid Benefit, *BAHU* BAHA Aesthetic, hygiene and Use, *COSI* Client Oriented Scale of Improvement, *GBI* Glasgow Benefit Inventory, *N/A* Not available, *PTA* Pure Tone Audiometry, *QoL*: Quality of Life, *SRT* Speech Reception Thresholds

The studies from Baker et al. (2015) [23] and Powel et al. (2015) [26] studied predominantly paediatric population and both observed greater improvement in mean aided thresholds (41 dB HL and 30.6 dB HL respectively) compared to other studies with predominantly adult population, such as the one from Briggs et al. (2015) [22] who found improvements of 18.4 dB HL. Similarly, SRT appeared to be better in the paediatric population. This can be explained by the thinner soft tissue and less attenuation of vibration in children.

### Functional outcomes

In the study by Gawecki et al. (2016) [19], both groups (Group A: bilateral mixed and conductive hearing loss, Group B: unilateral deafness) reported significant improvement in the Global score of the Abbreviated Profile of Hearing Aid Benefit (APHAB) (mean gain: total 23.5 %, Group A 21.4 %, Group B 26.4 %). Seventeen patients (85 %) reported that the aesthetic effect of the Attract was good or very good. Regarding the Glasgow Benefit Inventory (GBI), the mean total score for both groups was 29.6 (general subscale 40.3, social support 13.3, physical health 3.3). Similarly, in the studies by Iseri et al. (2014,2015) [9, 24], 97 % of patients completed the GBI and the mean score was 40.5 (General subscale 47.6, Social support 28.1, Physical health 23.9). Briggs et al. (2015) [22] found a significant improvement in the global score of the APHAB (p < 0.05]. Powell et al. (2015) [26] designed a new questionnaire (Bone Anchored Hearing Devices Questionnaire) taking into consideration the Entific medical systems questionnaire and the APHAB. Quality of life was improved in all 6 patients and their overall satisfaction on a scale from 0 (very dissatisfied) to 10 (very satisfied) was 9.7. In the study by Carr et al. (2015) [27] the overall satisfaction scores on GBI for those who were aided before implantation was 91 % and for those who were not previously aided was 82 %. Regarding the Client Oriented Scale of Improvement (COSI), 70 % of patients responded that they could hear in noisy environments 75 % to 95 % of the time and all of them agreed that the sound quality was good or very good. Finally, no functional results were presented in the remaining 2 studies [6, 25].

### Complications

**Fig. 4:**
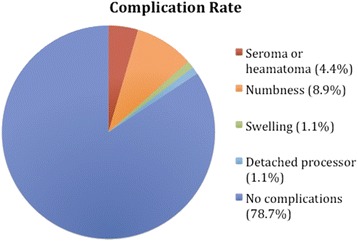
A pie chart that displays the complication rates of BAHA Attract implantation

A pie chart that displays the complication rates is presented in Fig. 4. Table 2 presents the complications per centre along with their management. A common problem reported amongst the studies is linked to the magnet strength; pain and erythema around the implant that resolve by lowering the magnet’s strength while weak attachment of the magnet is usually resolved by increasing the magnet strength. There is need to find the ideal balance between the two in each case. Seroma or haematoma formation was reported in 4 patients (4.4 %), which was treated conservatively [9, 19, 23]. Eight patients (8.9 %) reported to have numbness around the area of the flap in a single study [27]. This probably represents a commoner problem that is under-reported. One patient (1.1 %) was treated with antibiotics for a mild swelling that they developed 7 days post-operatively [25]. In another case (1.1 %) the sound processor would detach from the external magnet [26]. No major differences identified in the complications between the paediatric and adult patients. There have been no reports of persistent adverse reactions of skin due to the magnet up to this intermediate phase. However, it would be important to look out for any long-term complications of its use.

## Discussion

Strengths and Limitations of the studies and directions for future studies

A common limitation in the studies described is that they are observational studies (retrospective or prospective cohort studies) or case reports rather than Randomised Controlled Trials; therefore, confounding factors might have influenced reported outcomes. Some of the studies include a small number of patients so caution is needed in generalizing the results. Moreover, the outcome measures (Audiological and Functional) and the timing of testing varied greatly amongst the studies making a direct comparison difficult. Age and gender matching of participants in studies that compared different hearing solutions [9, 26] was not always done although this is hard to achieve in convenience studies. Finally, data on audiological, otological indications or functional outcomes were missing from some of the studies [2, 6, 9, 23, 25]. On the other hand, most studies had a good follow-up rate of their cohort and were able to present data for most of the participants. The manufacturers were not involved in the design, analysis or publication process of most studies. More specifically all but three studies disclosed no conflict of interest. Two studies [6, 23] did not declare any conflict of interest. The study by Briggs et al. (2015) [22] was sponsored by Cochlear Bone Anchored Solutions, Mölnlycke, Sweden.

In the future, more robust, well-designed studies with a higher level of evidence are needed. With rising healthcare costs and a demand for improving technology in an era of rationalization in healthcare, there is an ever-increasing need for hard evidence of the cost benefit ratio of new technology.

## Conclusions

This is the first systematic literature review on a new transcutaneous Bone Conduction Hearing Aid device, the BAHA Attract by the Cochlear Bone Anchored Solutions AB Mölnlycke, Sweden that was granted approval in 2013. Once the appropriate candidates have been selected through thorough evaluation the results have been promising. The surgery is relatively simple and quick and can be done under local anaesthesia or general anaesthesia. The functional and audiological results presented in the literature are quite satisfactory and the complication rate is much less compared to the skin penetrating BCD. A multi-centre randomized controlled trial that would test different hearing devices is currently missing from the literature.

## Abbreviations

APHAB: Abbreviated Profile of Hearing Aid Benefit
BAHA: Bone Anchored Hearing Aid
BCD: Bone conduction devices
CHL: Conductive hearing loss
COM: Chronic otitis media
COSI: Client Oriented Scale of Improvement
EAC: External auditory canal
ENT: Ear, nose and throat
GBI: Glasgow Benefit Inventory
MeSH: Medical subject headings
MRI: Magnetic resonance imaging
PTA: Pure Tone Audiometry
SRT: Speech recognition thresholds
SSD: Single sided deafness
WDS: Word discrimination scores
